# Adjuvant Activity of *Sargassum pallidum* Polysaccharides against Combined Newcastle Disease, Infectious Bronchitis and Avian Influenza Inactivated Vaccines

**DOI:** 10.3390/md10122648

**Published:** 2012-11-22

**Authors:** Li-Jie Li, Ming-Yi Li, Yan-Tuan Li, Jing-Jing Feng, Feng-Qiang Hao, Lun Zhang

**Affiliations:** 1 School of Medicinal and Pharmacy, Ocean University of China, 5 Yushan Road, Qingdao, Shandong 266003, China; 2 Shandong Sinder Technology Co., Ltd., Qingdao, Shandong 266061, China; Email: limingyia@yahoo.com.cn (M.-Y.L.); jingjingf123@126.com (J.-J.F.); haofengqiang1023@163.com (F.-Q.H.); zhanglun@sinder.cn (L.Z.)

**Keywords:** *Sargassum pallidum* polysaccharide, combined Newcastle disease, infectious bronchitis and avian influenza inactivated vaccines, antibody titer, T lymphocytes

## Abstract

This study evaluates the effects of *Sargassum pallidum* polysaccharides (SPP) on the immune responses in a chicken model. The adjuvanticity of *Sargassum pallidum* polysaccharides in *Newcastle disease* (ND), *infectious bronchitis* (IB) and *avian influenza* (AI) was investigated by examining the antibody titers and lymphocyte proliferation following immunization in chickens. The chickens were administrated combined ND, IB and AI inactivated vaccines containing SPP at 10, 30 and 50 mg/mL, using an oil adjuvant vaccine as a control. The ND, IB and AI antibody titers and the lymphocyte proliferation were enhanced at 30 mg/mL SPP. In conclusion, an appropriate dose of SPP may be a safe and efficacious immune stimulator candidate that is suitable for vaccines to produce early and persistent prophylaxis.

## 1. Introduction

Recent studies have shown that polysaccharides enhance the organism specificity and the non-specific immune function of vaccines [1,2,3,4,5,6]. Polysaccharides activate T cells, B cells, macrophages (Mφ), natural killer cells (NK), cytotoxic T cells (TL), lymphokine-activated killer cells (LAK) and other immune cells. Polysaccharides also promote the secretion of interleukin 1 (IL-1) and tumor necrosis factor (INF) from giant macrophages [7], the production of leukocyte interleukin 2 (IL-2) from T lymphocytes, the production of interferon (TFN) from activated white blood cells and the activation of the complement and reticuloendothelial systems in a variety of ways [8,9]. Polysaccharides can enhance immune function [10,11], have no toxic side effects in normal cells and are good biological response modifiers that can be developed into a new type of vaccine adjuvant [12,13]. Interest in these polysaccharides for the development of new adjuvants or immunopotentiators for medical and veterinary vaccines has increased [14,15,16,17]; however, the benefits of incorporating polysaccharides into combined Newcastle disease, infectious bronchitis and avian influenza inactivated vaccines have not been demonstrated. In our previous study, we determined that the *Sargassum* polysaccharide demonstrated a stronger potential to enhance serum antibody titers and lymphocyte proliferation in chickens compared with seven other Chinese herbal medicinal ingredients, such as *Astragalus* polysaccharide and *Lycium barbarum* polysaccharides.

Based on the above reasons, the immune-enhancing activities of *Sargassum pallidum *polysaccharides on combined Newcastle disease, infectious bronchitis and avian influenza inactivated vaccines were investigated in this study. The aim is to demonstrate the effectiveness and safety of *Sargassum pallidum* polysaccharides and to determine the optimum dose, which may provide theoretical evidence for the development of polysaccharide immunopotentiators.

## 2. Results

### 2.1. Changes in the Antibody Titer

#### 2.1.1. Changes in the ND Antibody Titer

**Figure 1:**
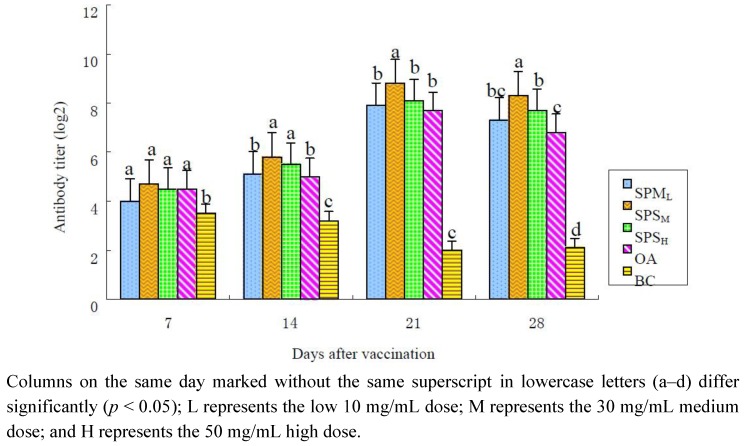
Changes in ND-HI antibody in each group in the immune response (log 2).

The changes in the ND-HI antibody titers are illustrated in Table 1 and Figure 1. After immunization, the antibody titers in the SPP groups (10, 30 and 50 mg/mL) and in the OA group at all time points were higher than those in the BC group, and the titers in the 30 mg/mL SPP group were the highest. The antibody titers in the 30 mg/mL SPP group were significantly higher than those in the OA group on the 14th, 21st and 28th days (*p* < 0.05). The antibody titers in the 50 mg/mL SPP group kept higher level than those in the OA group on the 14th and 28th days (*p* < 0.05).

**Table 1:** Dynamic changes in the Newcastle disease (ND) antibody titer in all groups.

Group	Days after the Immunization			
	7	14	21	28
SPP_L_	4.1 ± 0.40 ^a, b^	5.0 ± 0.27 ^b^	7.8 ± 0.25 ^b^	7.0 ± 0.30 ^b, c^
SPP_M_	4.9 ± 0.48 ^a^	6.1 ± 0.40 ^a^	8.8 ± 0.25 ^a^	8.6 ± 0.32 ^a^
SPP_H_	4.5 ± 0.19 ^a^	6.0 ± 0.19 ^a^	7.9 ± 0.23 ^b^	7.6 ± 0.38 ^b^
OA	4.6 ± 0.38 ^a^	4.9 ± 0.23 ^b^	7.6 ± 0.26 ^b^	6.5 ± 0.33 ^c^
BC	3.5 ± 0.19 ^b^	3.3 ± 0.41 ^c^	2.0 ± 0.27 ^c^	2.1 ± 0.13 ^d^

The data on the same day (columns) marked without the same superscript lowercase letters (a–d) differ significantly (*p* < 0.05); L represents the 10 mg/mL low SPP dose; M represents the 30 mg/mL medium SPP dose; and H represents the 50 mg/mL high SPP dose.

#### 2.1.2. Changes in the IB Antibody Titer

**Figure 2:**
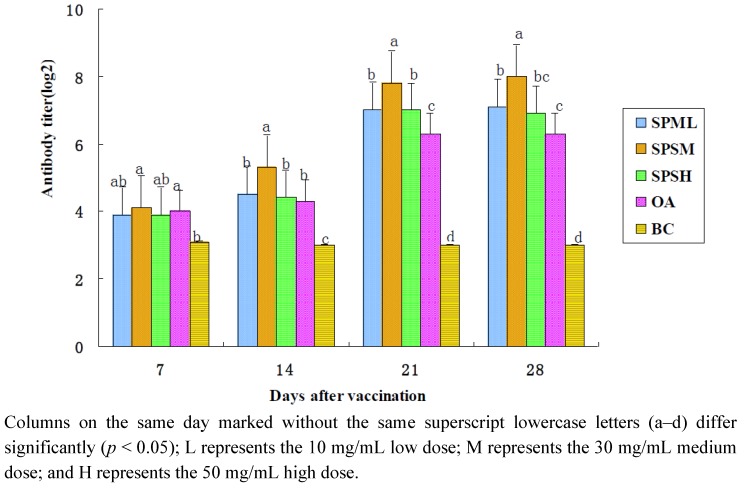
Changes in IB-HI antibody in each group in the immune response (log 2).

The changes in the IB-HI antibody titers are illustrated in Table 2 and Figure 2. After immunization, the antibody titers in the SPP groups (10, 30 and 50 mg/mL) and in the OA group at all time points were higher than those in the BC group, and the titers in the 30 mg/mL SPP group were the highest. The antibody titers in the 30 mg/mL SPP group were much higher compared with those in the OA group on the 14th, 21st and 28th day (*p* < 0.05). The antibody titers in the 10 mg/mL and 50 mg/mL SPP group were significantly higher than those in the OA group on the 21th and 28th day (*p* < 0.05).

**Table 2:** Dynamic changes in the *infectious bronchitis* (IB) antibody titer in all groups.

Group	Days after the Immunization			
	7	14	21	28
SPP_L_	3.9 ± 0.30 ^a, b^	4.5 ± 0.27 ^b^	7.0 ± 0.27 ^b^	7.1 ± 0.23 ^b^
SPP_M_	4.1 ± 0.23 ^a^	5.3 ± 0.25 ^a^	7.8 ± 0.25 ^a^	8.0 ± 0.27 ^a^
SPP_H_	3.9 ± 0.30 ^a, b^	4.4 ± 0.26 ^b^	7.0 ± 0.33 ^b^	6.9 ± 0.30 ^b, c^
OA	4.0 ± 0.27 ^a^	4.3 ± 0.25 ^b^	6.3 ± 0.25 ^c^	6.3 ± 0.25 ^c^
BC	3.1 ± 0.23 ^b^	3.0 ± 0.19 ^c^	3.0 ± 0.19 ^d^	3.0 ± 0.30 ^d^

The data on the same day (columns) marked without the same superscript lowercase letters (a–d) differ significantly (*p* < 0.05); L represents the 10 mg/mL low SPP dose; M represents the 30 mg/mL medium SPP dose; and H represents the 50 mg/mL high SPP dose.

#### 2.1.3. Changes in the AI Antibody Titer

**Figure 3:**
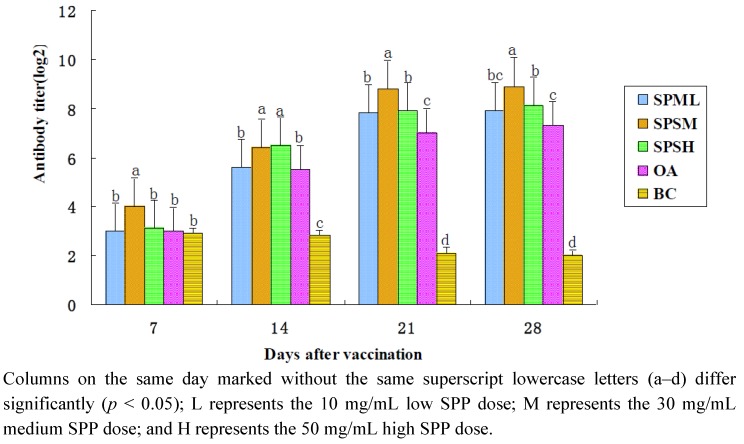
Changes in AI-HI antibody in each group in the immune response (log 2).

The changes in the AI-HI antibody titers are illustrated in Table 3 and Figure 3. After immunization, the antibody titers in the 30 mg/mL SPP group were significantly higher compared with those in the OA group and the BC group at all time points (*p* < 0.05). The antibody titers in the OA group were lower than those in the 50 mg/mL SPP group on the 14th, 21st and 28th days (*p* < 0.05) and those in the 10 mg/mL SPP group on the 21st day (*p* < 0.05).

**Table 3:** Dynamic changes in the *avian influenza* (AI) antibody titer in all groups.

Group	Days after the Immunization			
	7	14	21	28
SPP_L_	3.0 ± 0.27 ^b^	5.6 ± 0.18 ^b^	7.8 ± 0.25 ^b^	7.9 ± 0.30 ^b, c^
SPP_M_	4.0 ± 0.27 ^a^	6.4 ± 0.32 ^a^	8.8 ± 0.16 ^a^	8.9 ± 0.23 ^a^
SPP_H_	3.1 ± 0.30 ^b^	6.5 ± 0.19 ^a^	7.9 ± 0.30 ^b^	8.1 ± 0.23 ^b^
OA	3.0 ± 0.76 ^b^	5.5 ± 0.27 ^b^	7.0 ± 0.27 ^c^	7.3 ± 0.25 ^c^
BC	2.9 ± 0.23 ^b^	2.8 ± 0.16 ^c^	2.1 ± 0.30 ^d^	2.0 ± 0.27 ^d^

The data on the same day (columns) marked without the same superscript lowercase letters (a–d) differ significantly (*p* < 0.05); L represents the 10 mg/mL low SPP dose; M represents the 30 mg/mL medium SPP dose; and H represents the 50 mg/mL high SPP dose.

### 2.2. Changes in the Lymphocyte Proliferation Assay

The changes in lymphocyte proliferation (*A*_570_ value) are shown in Table 4. After immunization, the *A*_570_ value in the SPP adjuvant (10, 30 and 50 mg/mL) groups and in the OA group were significantly higher than those in the BC group (*p* < 0.05) at all the time points. The *A*_570_ value in the 30 mg/mL and 50 mg/mL SPP group kept higher level than those in the OA and BC groups at all the time points (*p* < 0.05). The *A*_570_ value in the 10 mg/mL SPP group was significantly higher than that in the OA group on the 14th, 21st and 28th days (*p* < 0.05).

**Table 4:** Dynamic changes in T lymphocyte proliferation in all groups (*A*_570_ value).

Group	Days after the Immunization			
	7	14	21	28
SPP_L_	0.241 ± 0.088 ^b^	0.279 ± 0.025 ^b^	0.402 ± 0.049 ^c^	0.395 ± 0.073 ^c^
SPP_M_	0.260 ± 0.026 ^a^	0.295 ± 0.013 ^a^	0.449 ± 0.042 ^a^	0.526 ± 0.017 ^a^
SPP_H_	0.257 ± 0.041 ^a^	0.282 ± 0.015 ^b^	0.417 ± 0.062 ^b^	0.477 ± 0.047 ^b^
OA	0.234 ± 0.035 ^b^	0.257 ± 0.017 ^c^	0.354 ± 0.029 ^d^	0.382 ± 0.026 ^d^
BC	0.213 ± 0.010 ^c^	0.188 ± 0.009 ^d^	0.201 ± 0.024 ^e^	0.158 ± 0.086 ^e^

The data on the same day (columns) marked without the same superscript lowercase letters (a–e) differ significantly (*p* < 0.05); L represents the 10 mg/mL low SPP dose; M represents the 30 mg/mL medium SPP dose; and H represents the 50 mg/mL high SPP dose.

### 2.3. Changes in the Lymphocyte Subsets

#### 2.3.1. Changes in CD_4_^+^ Lymphocyte Subsets

The changes in the CD_4_^+^ lymphocyte subsets (*A*_570_ value) are shown in Table 5. After immunization, the proportion of CD_4_^+^ T lymphocytes in the SPP adjuvant (10, 30 and 50 mg/mL) and OA groups was significantly higher than that in the BC group (*p* < 0.05) at all the time points. The 30 mg/mL SPP group contained the highest proportion of CD_4_^+^ T lymphocytes. The proportion of CD_4_^+^ T lymphocytes in the 50 mg/mL SPP group was much higher compared with that in the OA group at all the time points (*p* < 0.05).

**Table 5:** Dynamic changes of CD_4_^+^ T lymphocyte subsets in every group (%).

Group	Days after the Immunization			
	7	14	21	28
SPP_L_	34.9 ± 3.84 ^c^	36.4 ± 3.71 ^b, c^	36.9 ± 3.74 ^c^	39.8 ± 3.70 ^b^
SPP_M_	39.3 ± 4.55 ^a^	40.2 ± 4.23 ^a^	40.3 ± 3.90 ^a^	42.5 ± 4.18 ^a^
SPP_H_	36.9 ± 4.11 ^b^	37.7 ± 4.02 ^b^	38.3 ± 3.79 ^b^	39.0 ± 4.05 ^b^
OA	34.6 ± 3.95 ^c^	35.0 ± 3.86 ^c^	36.7 ± 4.32 ^c^	37.5 ± 3.93 ^c^
BC	29.2 ± 4.56 ^d^	27.4 ± 3.80 ^d^	28.0 ± 3.54 ^d^	26.1 ± 3.61 ^d^

The data on the same day (columns) marked without the same superscript lowercase letters (a–d) differ significantly (*p* < 0.05); L represents the 10 mg/mL low SPP dose; M represents the 30 mg/mL medium SPP dose; and H represents the 50 mg/mL high SPP dose.

#### 2.3.2. Changes in CD_8_^+^ Lymphocyte Subsets

The changes in the CD_8_^+^ lymphocyte subsets (*A*_570_ value) are listed in Table 6. On the 14th day after immunization, the CD_8_^+^ T lymphocyte percentage in the 30 mg/mL SPP adjuvant was higher than that in the BC group. On the 21st day, the CD_8_^+^ T lymphocyte percentage in the three SPP adjuvant groups was higher than that in the BC group. The CD_8_^+^ T lymphocyte percentage in each experimental group were lower than those in the BC group at other time points; however, the difference was not significant (*p* > 0.05).

**Table 6:** Dynamic changes of CD_8_^+^ T lymphocyte subsets in every group (%).

Group	Days after the Immunization			
	7	14	21	28
SPP_L_	18.5 ± 2.15	20.8 ± 2.44	23.9 ± 1.41	22.8 ± 2.35
SPP_M_	19.7 ± 3.47	22.9 ± 2.27	23.6 ± 1.39	22.4 ± 2.12
SPP_H_	20.7 ± 2.64	21.2 ± 3.57	22.4 ± 2.89	21.8 ± 1.23
OA	19.4 ± 2.08	21.5 ± 1.62	21.9 ± 3.02	22.6 ± 3.27
BC	21.6 ± 1.48	21.5 ± 3.82	22.0 ± 3.31	22.8 ± 2.46

L represents the 10 mg/mL low SPP dose; M represents the 30 mg/mL medium SPP dose; and H represents the 50 mg/mL high SPP dose.

#### 2.3.3. The Changes in CD_4_^+^/CD_8_^+^ Values

The changes in the CD_4_^+^/CD_8_^+^ values (*A*_570_ value) are listed in Table 7. On the 7th day after immunization, the differences in the CD_4_^+^ / CD_8_^+^ values in each experimental group were significantly higher than those in the BC group (*p* < 0.05). On the 7th and 28th day, the CD_4_^+^/CD_8_^+^ ratio in the 30 mg/mL SPP adjuvant group was significantly higher than those in the other groups (*p* < 0.05). 

**Table 7:** Dynamic changes of the ratio of T lymphocyte CD_4_^+^/CD_8_^+^ in every group.

Group	Days after the Immunization			
	7	14	21	28
SPP_L_	1.80 ± 0.40 ^c^	1.75 ± 0.33 ^a^	1.57 ± 0.17 ^b, c^	1.73 ± 0.18 ^b, c^
SPP_M_	2.01 ± 0.35 ^a^	1.75 ± 0.12 ^a^	1.73 ± 0.29 ^a^	1.85 ± 0.37 ^a^
SPP_H_	1.91 ± 0.28 ^b^	1.74 ± 0.17 ^a^	1.74 ± 0.34 ^a^	1.79 ± 0.16 ^b^
OA	1.73 ± 0.14 ^c, d^	1.68 ± 0.09 ^b^	1.64 ± 0.50 ^b^	1.66 ± 0.45 ^c^
BC	1.34 ± 0.15 ^e^	1.29 ± 0.26 ^c^	1.25 ± 0.41 ^d^	1.12 ± 0.22 ^d^

The data from the same day (column) marked without the same superscript lowercase letters (a–d) differ significantly (*p* < 0.05); L represents the 10 mg/mL low dose; M represents the 30 mg/mL medium dose; and H represents the 50 mg/mL high dose.

## 3. Experimental

### 3.1. Preparation of Polysaccharides

*Sargassum pallidum* was purchased from Qingdao Hai-jie Aquatic Science and Technology Co., Ltd., Shandong, China. The *Sargassum pallidum* polysaccharides (SPP) are polysaccharides compound easily dissolve in water, high viscosity, which are extracted from *Sargassum pallidum*.Ye analyse monosaccharide composition of SPP as rhamnose, xylose, fucose, mannose, glucose and galactose by GC. Polysaccharides in the *Sargassum pallidum *include outermost skeleton polysaccharide, the intercellular mucopolysaccharide and storage polysaccharides in protoplasts. The recent studies about SPP in the domestic and foreign are the intercellular mucopolysaccharide primarily [18,19]. The SPP we used were prepared as previously described [20]. The net polysaccharide content was 81.2%, as measured using the sulfuric acid-anthrone method. The SPP were diluted with deionized water, sterilized and assayed for endotoxin using the pyrogen test. When the endotoxin content was at the level recommended by the standards of the Chinese Veterinary Pharmacopoeia (less than 0.5 EU/mL) [21], the SPP were stored at 4 °C.

### 3.2. Vaccine and Virus

The ND virus vaccine (La Sota strain, EID50, 8.5) and the IB virus vaccine (M41 strain, EID50, 6.5) were provided by the China Institute of Veterinary Drugs Control; the AI virus vaccine (H9, K strain, EID50, 7.5) was provided by Heilongjiang Baizhou Bio-engineering Co., Ltd. (Baizhou, China), and was identified by the Virus Influenza Centre of the Chinese Academy of Preventive Medicine. The SPP adjuvant vaccines were produce by mixing the vaccines with three different concentrations of SPP; the non-adjuvant vaccine was produced by diluting the vaccines with PBS, and the oil adjuvant vaccine was produced by emulsifying the vaccines with white oil, in accordance with a previous report [22]. All of the vaccines contained the same amount of antigen.

### 3.3. Reagents

Modified RPMI-1640 medium (Thermo Fisher Biochemical Products Co., Ltd., Beijing, China) supplemented with benzylpenicillin (100 IU/mL), streptomycin (100 IU/mL) and 10% (volume fraction) fetal bovine serum (Hangzhou Evergreen Biological Engineering Materials Co., Ltd., Hangzhou, China, No. 090610) was used for washing, re-suspending and culturing the cells and was stored at 4 °C. The concanavalin globulin (Con A; Sigma company, Beijing, China) was prepared at a concentration of 0.025 mg/mL in serum RPMI 1640 culture medium, was filter sterilized, dispensed and stored at −20 °C. The 3-(4,5-dimethyithiazol-2-yl)-2,5-diphenyltetrazolium bromide (MTT) kit, from the Institute of Biotechnology, was stored at 4 °C in the dark. The lymphocyte separation medium (No. 20110923) was purchased from Beijing Soledad Bao Technology Co., Ltd. (Beijing, China). The FITC-labeled rat anti-chicken CD3, PE-labeled rat anti-chicken CD4, PE-labeled rat anti-chicken CD8a, FITC-labeled rat anti-chicken IgGl, and PE-labeled rat anti-chicken IgGl monoclonal antibodies were obtained from the Jingmei Biotech Co., Ltd. (Shenzhen, China).

### 3.4. Animals and Immunization

The AA broiler chickens, one day old, were purchased from the Zhucheng Foreign Trade Breeder Farm, and were housed in an isolator (220 cm × 85 cm × 180 cm) in an air-conditioned room at 37 °C with light for 24 h per day at the beginning of the pretest period. The temperature was gradually decreased to room temperature, and the light time was gradually decreased to 12 h per day; these conditions were maintained until sacrifice. The chickens were fed with a commercial starter diet from the Qingdao Zhengda Agricultural Development Co., Ltd. (Qingdao, China). All the procedures were performed in strict accordance with internationally accepted principles and the *PR China* legislation on the use and care of laboratory animals.

The chickens were divided randomly into 5 groups, with 50 chickens per group (250 in total). The chickens were 14 days old, the average maternal ND antibody titer was 4.6 log 2, the IB antibody titer was 3.8 log 2, the AI antibody titer was 4.3 log 2, and the average body weight was 232 g. The chickens were subcutaneously injected with the vaccines containing 10, 30 or 50 mg/mL of the SPP adjuvant, the oil adjuvant (OA), or physiological concentrations of saline (blank control group, BC). 

### 3.5. Serum Antibody Titer Assay [23,24]

#### 3.5.1. Serum ND Antibody Titer Assay

On days 7, 14, 21, and 28, after vaccination, 10 chickens were sampled randomly from each group for the determination of the ND-HI antibody titer. Serial dilutions (2-fold) of serum were placed in the V-shaped bottom 96-well microtiter plate containing 25 μL of CMF-PBS in each well; next, 25 µL of antigen (NDV, IBV or AIV, 4 HA units) was added to the wells with the exception of the last row, which served as the control wells. The serum dilutions ranged from 1:2 to 1:2048. The antigen-serum mixture was incubated for 10 min at 37 °C. Next, 25 µL of a 1% rooster erythrocyte suspension was added to each well and the plate was incubated for 30 min. The control wells were positive for serum, negative for serum, contained erythrocytes, or contained antigens. The highest dilution of serum that caused complete inhibition was considered the endpoint. The geometric mean titer was expressed as the reciprocal log 2 values of the highest dilution that exhibited HI [25,26].

#### 3.5.2. Serum IB Antibody Titer Assay

On days 7, 14, 21, and 28, after vaccination, 10 chickens were sampled randomly from each group for the determination of IB-HI antibody titer as described in Section 3.5.1.

#### 3.5.3. Serum AI Antibody Titer Assay

On days 7, 14, 21, and 28, after vaccination, 10 chickens were sampled randomly from each group for the determination of AI-HI antibody titer as described in Section 3.5.1.

### 3.6. Peripheral Blood Lymphocyte Proliferation Assay

On days 7, 14, 21, and 28, after vaccination, 6 chickens were used to determine the proliferation of peripheral lymphocytes using the MTT assay [27]. Whole blood samples were collected from the immune chickens and were immediately transferred into aseptic capped tubes containing sodium heparin, which was then diluted with an equal volume of Hanks solution and was carefully layered onto the surface of lymphocyte separation medium. After centrifugation at 800× *g* for 20 min, the cloud-like lymphocyte band was collected and washed twice with RPMI-1640 media without fetal bovine serum. The resulting pellet was re-suspended at 5.0 × 106 cells/mL in improved RPMI-1640 medium containing 10% fetal bovine serum. The cells were added to 96-well flat-bottomed cell culture plates (50 µL per well), and ConA (50 µL, final concentration of 10 μg/mL) was added. Each sample was prepared in triplicate, and the cells grown without ConA were used as the negative control. The plates were incubated in a humidified 5% CO_2_ incubator (HF160W, Heal Force Bio-Meditech Holdings Limited., Shanghai, China) at 39 °C for 48 h. The MTT solution (10 µL) was added to each well and the incubation was continued until the wells were covered with deep-purple-colored crystalline formanzan. Formanzan-dissolving liquid (100 µL) was added to each well, and the incubation was continued in the CO_2_ incubator until the formanzan was dissolved (visualized using a CKX41 OLYMPUS microscope, OLYMPUS (China) Co., Ltd., Shanghai, China). The plate was read at 570 nm (*A*_570_ value) using a multi-well absorbance reader (Model 680, BIO-RAD, Shanghai, China).

### 3.7. Flow Cytometry Analysis

On days 7, 14, 21, and 28 after vaccination, 6 chickens were used to determine the changes in the lymphocytes subsets. The lymphocytes were separated as described in Section 3.6. The cell number was adjusted to 5.0 × 106 cells/mL with PBS solution. FITC-labeled rat anti-chicken CD3 (10 µL) and PE-labeled rat anti-chicken CD4 (10 µL) were added to one sample, FITC-labeled rat anti-chicken CD3 (10 µL) and PE-labeled rat anti-chicken CD8a (10 µL) were added to a second sample, and FITC-labeled rat anti-chicken IgGl (10 µL) and PE-labeled rat anti-chicken IgGl (10 µL) were added to the control sample. The samples were added to the lymphocyte suspension (50 µL) and were mixed in the dark at 4 °C for 20 min. Next, PBS (500 µL) was added and the samples were mixed in the dark at 25 °C for 10 min. The cells were re-suspended and analyzed using a FACSCaliburTM flow cytometer (BD Bioscience, Beijing, China) [28].

### 3.8. Statistical Analysis

Data analysis was performed with SPSS 16.0 software. One-Way ANOVA with Duncan’s post hoc test was used for multiple comparisons between groups. Values were expressed as the mean ± standard deviation (SD). The results of the comparisons between groups were considered significantly different if *p* < 0.05.

## 4. Discussion

The humoral response is mediated by the secretion of immune antibodies by B lymphocytes. The antibody titer is a measure of the specific humoral immune function in chickens after vaccination. In present study, higher antibody titers were found in *Sargassum pallidum* polysaccharides immune group on days 7, 14, 21 and 28 after the vaccination, especially in the 30 mg/mL SPP group. The results provided evidences that SPP could enhance the humoral response in chickens and the immune effect of the medium doses of SPP was more effective than the high doses. This observation corresponds with a report by Wang [29] showing the effectiveness of Chinese herbal medicine compounds in enhancing immunity in chickens. The reason is that adding high doses of polysaccharide to vaccines increases the volume of the vaccine at the injection site and makes it difficult to absorb, which affects the immune effect.

CD_4_ and CD_8_ are important T lymphocyte markers; the main function of CD_4_^+^ T lymphocytes is the secretion of cell factors that induce and enhance the immune response, and CD_8_^+^ T lymphocytes mainly mediate cytotoxic effects [30]. CD_4_^+^ and CD_8_^+^ T lymphocytes are the hub of immune regulation, and when the CD_4_^+^/CD_8_^+^ ratio is high but within the normal range, the body has a high immune status, whereas when the CD_4_^+^/CD_8_^+^ ratio is disrupted, this can lead to a variety of immune diseases. The results of T subsets showed that the vaccination of the groups containing SPP (especially 30 mg/mL SPP) may lead to the polarization of cell responses within 7 days. These findings indicate that the SPP could significantly enhance cellular immunity, which in turn could activate the antibody response.

The SPP exhibit an immunity adjustment function by affecting T and B lymphocyte multiplication. However, because SPP are composed of many monosaccharide molecules, such as glucose, rhamnose, and arabinose, differences in the compositions of various polysaccharides are seldom studied and the mechanisms of immune response enhancement are not clear. The mechanism of the regulatory role of the immune function may be as follows: (1) SPP induce the production of cytokines by stimulating non-specific immune cells (macrophages or natural killer cells) to activate the T and B immune cells; (2) unknown components of the SPP may contain a type of cytokine that binds to cell surface receptors to promote T and B cell differentiation and proliferation; (3) a number of small lipid-soluble polysaccharides from SPP may directly enter T and B lymphocyte and affect cell metabolism or the secretion of cytokines, among other factors [31].

## 5. Conclusion

The effects of SPP against the combined ND-IB-AI inactivated vaccines on the immune responses of chicken have been studied. From this study, the following conclusions are derived:

1. The results of this study show that the appropriate dose of polysaccharide significantly enhances the specific immune response in chickens and improves vaccine effectiveness, promoting an earlier peak that increases rapidly and lasts for a long time;
2. The lymphocyte transformation state corresponds to the *A*_570_ values measured using the MTT method. The data indicate that the SPP induce T lymphocytes to multiply and exhibit certain effectiveness;
3. In this study, the CD_4_^+^ T lymphocyte content and CD_4_^+^/CD_8_^+^ values in all the test groups were higher than those in the control group, and the medium SPP dose groups were significantly higher than the control group, which indicates that the appropriate dose of polysaccharide can promote the proliferation of peripheral CD_4_^+^ T lymphocytes in chickens, thereby enhancing cellular immunity;
4. Co-administration of SPP induced an increase in the proliferation rate and antibody production in lymphocytes, and an appropriate dose of SPP up-regulated both the cellular and humoral immune responses when used as an adjuvant for the combined ND-IB-AI vaccines.
